# Functional Expression of Programmed Death-Ligand 1 (B7-H1) by Immune Cells and Tumor Cells

**DOI:** 10.3389/fimmu.2017.00961

**Published:** 2017-08-10

**Authors:** Rachel M. Gibbons Johnson, Haidong Dong

**Affiliations:** ^1^Biology Discipline, University of Minnesota Morris, Morris, MN, United States; ^2^Department of Urology, College of Medicine, Mayo Clinic, Rochester, MN, United States; ^3^Department of Immunology, College of Medicine, Mayo Clinic, Rochester, MN, United States

**Keywords:** immunotherapy, programmed death-1:programmed death-ligand 1 blockade, B7-H1 (programmed death-ligand 1), T cells, CTL, tumor

## Abstract

The programmed death-1 (PD-1) and its ligand PD-L1 (B7-H1) signaling pathway has been the focus of much enthusiasm in the fields of tumor immunology and oncology with recent FDA approval of the anti-PD-1 antibodies pembrolizumab and nivolumab and the anti-PD-L1 antibodies durvalumab, atezolimuab, and avelumab. These therapies, referred to here as PD-L1/PD-1 checkpoint blockade therapies, are designed to block the interaction between PD-L1, expressed by tumor cells, and PD-1, expressed by tumor-infiltrating CD8^+^ T cells, leading to enhanced antitumor CD8^+^ T cell responses and tumor regression. The influence of PD-L1 expressed by tumor cells on antitumor CD8^+^ T cell responses is well characterized, but the impact of PD-L1 expressed by immune cells has not been well defined for antitumor CD8^+^ T cell responses. Although PD-L1 expression by tumor cells has been used as a biomarker in selection of patients for PD-L1/PD-1 checkpoint blockade therapies, patients whose tumor cells lack PD-L1 expression often respond positively to PD-L1/PD-1 checkpoint blockade therapies. This suggests that PD-L1 expressed by non-malignant cells may also contribute to antitumor immunity. Here, we review the functions of PD-L1 expressed by immune cells in the context of CD8^+^ T cell priming, contraction, and differentiation into memory populations, as well as the role of PD-L1 expressed by tumor cells in regulating antitumor CD8^+^ T cell responses.

## Introduction

Programmed death-ligand 1 (PD-L1, also referred to as B7-H1 or CD274) is constitutively expressed by cells of the myeloid lineage, including macrophages and dendritic cells (1–3). Cells from the lymphoid, endothelial, and epithelial lineages, including cancer cells from these lineages, express PD-L1 upon activation by IFN-γ and TNF-α (4). Naïve murine T cells express low levels of PD-L1, while naïve human T cells do not; however, both murine and human T cells express high levels of PD-L1 upon antigen stimulation (3, 5). PD-L1 has been extensively characterized as a ligand for PD-1, an inhibitory receptor expressed by activated CD8^+^ T cells. Upon interaction with PD-L1, PD-1 counteracts signaling downstream of T cell receptor (TCR) ligation and CD28 co-stimulation (6). When PD-L1 interacts with PD-1, the immunoreceptor tyrosine-based inhibitory motifs and immunoreceptor tyrosine-based switch motifs on the intracellular domain of PD-1 become phosphorylated. This recruits the phosphatases SHP-1 and SHP-2 to the intracellular domain of PD-1, which is in the vicinity of the TCR. SHP-1 and SHP-2 dephosphorylate the immunoreceptor tyrosine-based activation motifs of the TCR, thus dampening the signaling downstream of the TCR (7). By inhibiting TCR signaling, PD-1 prevents the activation of the PI3K/Akt and c-Myc pathways, and inhibits cell survival, proliferation, and cytokine production by CD8^+^ T cells (8). It is important to note that PD-L1 also interacts with CD80 (B7-1), which is expressed on the surface of CD8^+^ T cells. The signaling events that are initiated downstream of CD80 are still under investigation, but have been shown to have similar effects on CD8^+^ T cell function as signaling downstream of the PD-L1/PD-1 interaction (9–11).

The rationale for PD-L1/PD-1 checkpoint blockade therapies is to block the PD-L1/PD-1 interaction between tumor cells and CD8^+^ T cells with an antibody to allow for CD8^+^ T cells to overcome PD-1 inhibitory signaling and eliminate the tumor cells. FDA-approved anti-PD-1 antibodies include pembrolizumab and nivolumab. Currently pembrolizumab is approved for treatment of metastatic melanoma, both squamous and non-squamous non-small cell lung cancer (NSCLC), head and neck squamous-cell carcinoma, and Hodgkin’s lymphoma, and nivolumab is approved for treatment of metastatic melanoma, both squamous and non-squamous NSCLC, and renal-cell carcinoma. FDA-approved anti-PD-L1 antibodies include durvalumab, atezolimuab, and avelumab. Currently, durvalumab is approved for treatment of urothelial carcinoma, atezolimuab is approved for treatment of NSCLC and urothelial carcinoma, and avelumab is approved for treatment of Merkel cell carcinoma. PD-L1/PD-1 checkpoint blockade treatments yield durable responses for a significant number of patients, but many patients exhibit variable responses or no response to the treatments (12–24). These drugs are all in ongoing clinical trials to determine their use for additional tumor types.

There are also efforts underway to identify biomarkers that can be used to predict which patients will likely respond to PD-L1/PD-1 checkpoint blockade therapies. Logically, the expression of PD-L1 by a patient’s tumor cells is the focus of much of these efforts and will be discussed below. Numerous other biomarkers have been identified as useful tools for distinguishing “non-responders” from “responders” for PD-L1/PD-1 checkpoint blockade therapy. The presence of circulating PD-1^+^ CD8^+^ T cells that express the pro-apoptotic molecule Bim was predictive of clinical benefit in patients with metastatic melanoma that were treated with anti-PD-1 checkpoint blockade therapy (25). When PD-1^+^ CD8^+^ T cells interact with PD-L1, the expression of Bim is increased (11), therefore Bim expression by circulating PD-1^+^ CD8^+^ T cells is associated with CD8^+^ T cells that have encountered PD-L1 and would be reinvigorated by PD-L1/PD-1 checkpoint blockade. PD-1^+^ CD8^+^ T cells that are reinvigorated by PD-L1/PD-1 checkpoint blockade depend on CD28 co-stimulation for proliferation, thus the expression of CD28 by circulating PD-1^+^ CD8^+^ T cells has also been identified as a potential biomarker for predicting responses to PD-L1/PD-1 checkpoint blockade therapy (26). PD-L1 is classically characterized as membrane-bound, but a soluble form of PD-L1 (sPD-L1) also exists and high serum levels of sPD-L1 are associated with poor prognosis in renal-cell carcinoma and multiple myeloma (27, 28). Increased levels of sPD-L1 in the circulation of patients treated with PD-L1/PD-1 checkpoint blockade for malignant melanoma predicted a greater chance of partial response to therapy when assayed at 5 months after initiation of treatment (29). Tumors with deficiency in the mismatch-repair pathway for DNA repair or with high microsatellite instability are highly immunogenic and patients with tumors having either of these characteristics are more likely to respond to PD-L1/PD-1 checkpoint blockade therapy (30). These types of tumors, regardless of tissue of origin, generate multitudes of novel antigens due to their high mutation burden, making them ideal targets for CD8^+^ T cell responses reinvigorated by PD-L1/PD-1 checkpoint blockade therapy. The FDA recently approved pembrolizumab as a treatment for any unresectable or metastatic solid tumor that is mismatch repair deficient or has high microsatellite instability. This is the first FDA-approved treatment that is based on a biomarker rather than on the tissue of origin of the tumor.

As mentioned earlier, PD-L1 expression by tumor cells has been characterized as a biomarker for predicting patient outcomes to PD-L1/PD-1 checkpoint blockade therapy. Multiple studies have shown that PD-L1 expression is predictive of positive responses to PD-L1/PD-1 checkpoint blockade therapy (31–33). However, patients with PD-L1 negative tumors often exhibit positive responses to PD-L1/PD-1 checkpoint blockade therapies (20). Despite these findings, the FDA has approved PD-L1 biomarker tests as companions to nivolumab for treatment of NSCLC, pembrolizumab for treatment of NSCLC, and atezolimuab for treatment of urothelial carcinoma. Some of the unpredictability of responses to PD-L1/PD-1 checkpoint blockade therapies may be due to the heterogeneity and dynamics of PD-L1 expression by tumor cells and immune cells, including dendritic cells and CD8^+^ T cells (34, 35). Recent reports indicate that PD-L1 expressed by both tumor cells and non-tumor host cells contributes to the efficacy of PD-L1/PD-1 checkpoint blockade therapies in preclinical models (36, 37). It is essential to consider the role of the PD-L1/PD-1 interaction outside of the tumor setting in order to gain a better understand of the effects of PD-L1/PD-1 checkpoint blockade therapies.

**Figure 1:**
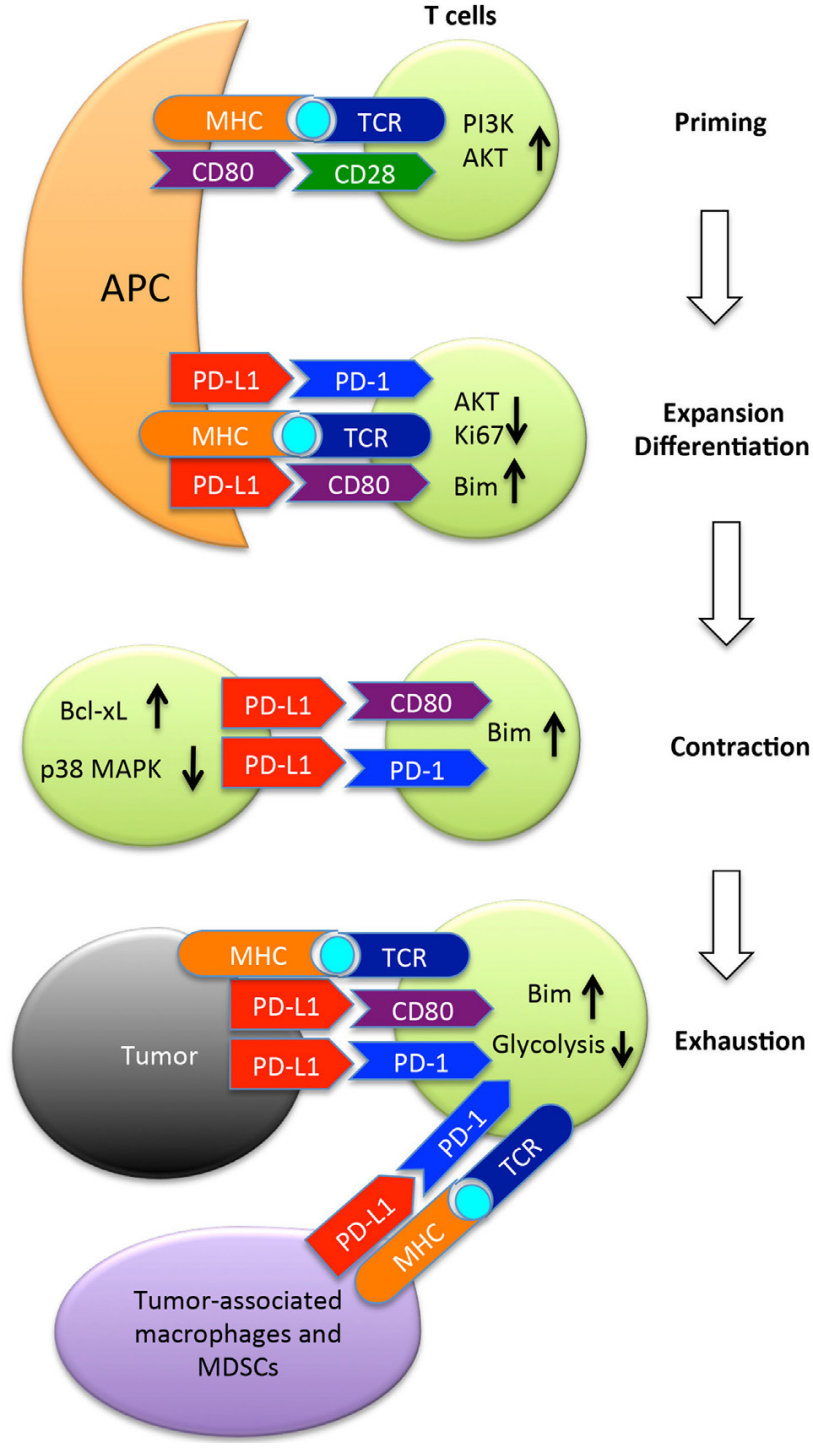
The programmed death ligand-1 (PD-L1) (B7-H1) pathway in regulation of T cell responses to tumors. Once naïve T cells encounter cognate antigen presented by antigen-presenting cells (APC), they are primed and express PD-1 and CD80, two receptors of PD-L1. PD-1 once engaged by its ligand recruits the phosphatase SHP-2 to inhibit AKT activation by preventing CD28-mediated activation of PI3K. Along with CD80, PD-1 suppresses T cell expansion, and limits differentiation to memory cells by upregulation of Bim. Following expansion, T cells undergo contraction in which PD-L1 expressed by activated T cells provides pro-survival signals to T cells by upregulating Bcl-xl and inhibiting p38 MAPK activation. After arriving at the tumor site, PD-L1 expressed by tumor cells or tumor-associated macrophages or MDSCs suppresses the function of tumor-reactive T cells by inducing either apoptosis or exhaustion. MDSC, myeloid-derived suppressor cells; Bim, Bcl-2-like protein 11.

The PD-L1/PD-1 interaction is involved during the initial activation or priming phase of naïve CD8^+^ T cells as PD-L1 is expressed on the surface of dendritic cells in the spleen and lymph nodes where T cell priming takes place, and PD-1 expression by CD8^+^ T cells is upregulated early on during the priming phase (38–40). In this review, we will consider how PD-L1/PD-1 checkpoint blockade therapies affect the priming phase of antitumor CD8^+^ T cell responses. Activated CD8^+^ T cells also express PD-L1, and it has been demonstrated that this expression of PD-L1 plays a role in the survival of activated CD8^+^ T cells during the contraction phase of an immune response (41, 42). Accordingly, the CD8^+^ T cell intrinsic role for PD-L1/PD-1 signaling will also be examined. Finally, the role of PD-L1 expressed by tumor cells in regulating CD8^+^ T cell antitumor immune responses will be considered. These interactions are summarized in Figure 1.

## The Role of PD-L1 Expressed by Dendritic Cells

Both PD-1 and CD80 are expressed on CD8^+^ T cells early during activation and priming by dendritic cells (43–45). Additionally, dendritic cells express cell-surface PD-L1 upon activation by various toll-like receptor ligands (40). In considering how PD-L1/PD-1 checkpoint blockade therapies influence antitumor CD8^+^ T cell responses, it is important to first consider the role of PD-L1 during the priming phase of an immune response. During this phase, naïve CD8^+^ T cells that are reactive against tumor antigens get activated in secondary lymphoid organs and undergo important changes in differentiation that will affect the resulting effector and memory antitumor CD8^+^ T cell responses. Several studies have demonstrated that PD-L1 signaling during the priming phase limits CD8^+^ T cell responses, so PD-L1/PD-1 checkpoint blockade therapies likely work in part by enhancing CD8^+^ T cell responses by influencing events during the priming phase of an antitumor immune response.

In order to generate an effective antitumor CD8^+^ T cell response, the signals provided by dendritic cells during the priming phase must initiate rapid proliferation of antitumor CD8^+^ T cells as well as orchestrate differentiation programs that will give rise to effector CD8^+^ T cells and memory CD8^+^ T cells. Several groups have independently demonstrated that CD8^+^ T cells exhibit increased proliferation when primed in the absence of PD-L1 signaling, indicating that PD-L1 signaling serves to restrict the proliferative capacity of CD8^+^ T cells during activation. Benedict et al. demonstrated that dendritic cells infected with CMV had high surface expression of PD-L1 and were unable to induce proliferation of antigen-specific CD8^+^ T cells. Including an antibody against PD-L1 in their *in vitro* priming model largely restored the ability of CMV-infected dendritic cells to induce proliferation of antigen-specific CD8^+^ T cells (46). In an *in vivo* priming model, we found that the numbers of antigen-specific CD8^+^ T cells significantly increased in animals immunized with activated dendritic cells that lacked PD-L1 expression as compared to activated dendritic cells with intact PD-L1 expression (40). Using an HSV-1 model, Channappanavar et al. demonstrated that systemic delivery of anti-PD-L1 antibody 1 day prior to HSV-1 infections allowed for increased proliferation of antigen-specific CD8^+^ T cells as compared to mice infected with HSV-1 in the absence of anti-PD-L1 treatment (47). Together these studies indicate that systemic treatment with PD-L1/PD-1 checkpoint blockade antibody therapy should result in increased proliferation of CD8^+^ T cell responses being primed in patients.

Differentiation of effector and memory CD8^+^ T cells occurs during the priming phase through a mechanism termed programming, in which naïve CD8^+^ T cells respond to external stimuli, including TCR signaling, co-stimulatory signaling, and cytokine signaling (38). The combination of these stimuli that a naïve CD8^+^ T cell encounters will determine the outcome of programming and have long-lasting impacts on the resulting effector and memory populations (48). In order to generate a potent effector and memory CD8^+^ T cell responses, naïve CD8^+^ T cells must encounter a cognate TCR stimulus in the context of positive co-stimulatory signals and pro-inflammatory cytokines (49). It has been well established that PD-L1 signaling is integrated during CD8^+^ T cell priming to restrain the differentiation of effector and memory CD8^+^ T cells.

Effector CD8^+^ T cells primed in the absence of PD-L1 signaling exhibit increased cytokine production and enhanced cytotoxic activity as compared to CD8^+^ T cells primed in the presence of PD-L1 signaling (40, 44, 45, 47, 50). Immunization of mice with PD-L1 deficient dendritic cells pulsed with OVA peptide resulted in effector CD8^+^ T cells that secreted increased levels of IFN-γ and were better able to control B16-OVA tumor growth as compared to effector CD8^+^ T cells primed by dendritic cells with intact PD-L1 expression (40). Similar results were found when anti-PD-L1 antibody was used to block PD-L1 signaling by the injected dendritic cells in this same study. CD8^+^ T cells activated in the absence of PD-L1 signaling had significantly increased production of IFN-γ (50). Using an HSV-1 infection model, Channappanavar et al. showed that blocking PD-L1 signaling during the priming phase resulted in effector CD8^+^ T cells with increased granzyme B exocytosis upon *ex vivo* antigen stimulation. Mice injected with anti-PD-L1 prior to HSV-1 infection also demonstrated significantly lower viral load 6 days postinfection (47). Using a brief *in vitro* priming model to activate OT-I CD8^+^ T cells with OVA-presenting dendritic cells with either intact or deficient PD-L1 expression, it was demonstrated that CD8^+^ T cells primed in the absence of PD-L1 secreted increased levels of IFN-γ and exhibited increased *in vivo* cytotoxic activity (45). These studies show that PD-L1 signaling during the priming phase influences the differentiation of effector CD8^+^ T cells by restraining the acquisition of effector functions.

During the priming phase, PD-L1 also controls differentiation of the resulting population of memory CD8^+^ T cells (51). In the same HSV-1 infection model as described above, Channappanavar et al. investigated the influence of PD-L1 signaling during priming on the resulting antigen-specific CD8^+^ T cell memory population. PD-L1 blocking antibody or isotype control antibody was injected 1 day prior and 3 days after HSV-1 infection. Mice were re-challenged with HSV-1 32 days after infection and CD8^+^ T cell recall responses were assayed on day 4 after re-infection. In mice that primed an anti-HSV-1 CD8^+^ memory T cell response in the absence of PD-L1 signaling, the memory recall response exhibited increased antigen-specific secretion of IFN-γ and granzyme B (47). Similar data were generated using an *in vivo* priming model and B16-OVA tumor challenge in which wild-type mice were injected with naïve OT-I CD8^+^ T cells (CD45.2^+^) and OVA-presenting activated bone marrow-derived dendritic cells that were either wild type or PD-L1 deficient. On day 30 after the injection of the naïve CD8^+^ T cells and activated dendritic cells, the mice were challenged with an intravenous injection of B16-OVA tumor cells. The recall response of the CD45.2^+^ T cells was analyzed 4 days after tumor challenge. In mice that had PD-L1-deficient dendritic cells during the priming phase, there was an increase in IFN-γ in the lungs as compared to mice that received dendritic cells with intact PD-L1 (45). Together these studies demonstrate that PD-L1 signaling is integrated during the priming phase and limits the differentiation of memory CD8^+^ T cell populations.

An additional level of regulation during T cell priming is ligand-induced TCR down-modulation, in which TCRs that have engaged with cognate peptide–MHC are internalized and downstream signaling is terminated (52, 53). Karwacz et al. investigated the influence of PD-L1/PD-1 signaling on the process of TCR down-modulation during priming. They knocked-down PD-L1 expression in bone marrow-derived dendritic cells using lentivirus-delivered short hairpin RNA and found that in the absence of PD-L1 signaling during the priming phase (both *in vitro* and *in vivo*) CD8^+^ T cells exhibited decreased TCR down-modulation as compared to CD8^+^ T cells primed by bone marrow-derived dendritic cells with PD-L1 expression intact (50). They went on to show that when PD-L1 is absent during priming, CD8^+^ T cells failed to upregulate the E3 ubiquitin ligase Cbl-b, which has been demonstrated to contribute to ligand-induced TCR down-modulation (54–56). These results were replicated when antibodies blocking either PD-L1 or PD-1 were included in their *in vitro* priming model, thus implicating a role for PD-L1 signaling in TCR down-modulation. In addition to membrane expression of PD-L1 by dendritic cells, a sPD-L1 produced by activated human dendritic cells may provide a non-proximal regulation of circulating PD-1^+^ or CD80^+^ T cells by dendritic cells (57). Altogether these studies indicate that blockade of PD-L1 signaling during the priming phase of CD8^+^ T cells would be beneficial for priming antitumor immune responses.

## The Role of PD-L1 Expressed by Effector CD8^+^ T Cells

CD8^+^ T cells upregulate expression of PD-L1 upon antigen stimulation, so it is important to consider how PD-L1/PD-1 checkpoint blockade therapies influence effector CD8^+^ T cells. Several studies have reported a T cell intrinsic pro-survival/antiapoptotic role for PD-L1 expressed by effector CD8^+^ T cells; therefore, it is likely that PD-L1/PD-1 checkpoint blockade therapies have deleterious effects on the functions of effector CD8^+^ T cells. Since the goal of PD-L1/PD-1 checkpoint blockade therapies is to enhance CD8^+^ T cell killing of tumor cells, it is essential that we more fully understand the role of PD-L1 expressed by effector CD8^+^ T cells.

Using an OVA protein immunization model of antigen stimulation in mice, Pulko et al. demonstrated that PD-L1 expressed by effector CD8^+^ T cells is required for their survival during the contraction phase of an immune response (41). Six days following immunization with OVA protein and poly(I:C), PD-L1-deficient CD8^+^ T cells exhibited increased contraction and enhanced susceptibility to being killed by other CD8^+^ T cells. Additionally, upon *in vitro* antigen stimulation, PD-L1-deficient CD8^+^ T cells expressed lower levels of Bcl-xL, an antiapoptotic molecule. Saha et al. demonstrated similar findings in a mouse graft-versus-host disease model (58). PD-L1-deficient allogeneic donor CD8^+^ T cells exhibited decreased expansion and survival 5 days after transfer as compared to wild-type allogeneic donor CD8^+^ T cells. The impaired survival of PD-L1-deficient CD8^+^ T cells was determined to be due to lower expression levels of the pro-survival proteins Bcl-xL and CD127. Additionally, PD-L1-deficient allogeneic donor CD8^+^ T cells had diminished cytokine production and impaired metabolic activity, including decreased glycolytic capability, decreased oxidative phosphorylation, and decreased fatty acid oxidation as compared to wild-type allogeneic donor CD8^+^ T cells. Another report confirmed the decreased expression of Bcl-xL in PD-L1-deficient CD8^+^ T cells and proposed that the interaction of PD-L1 with CD80, both expressed by CD8^+^ T cells, contributes to CD8^+^ T cell survival and expansion (59). This pro-survival/antiapoptotic role for PD-L1 expressed by CD8^+^ T cells could explain the deleterious effects of PD-L1 blockade as reported in *Listeria monocytogenes* infection models (60–62) and could also explain the variable patient responses to PD-L1/PD-1 checkpoint blockade therapy. The role of PD-L1 expressed by CD8^+^ T cells within a tumor setting needs to be further elucidated. It was recently demonstrated that certain anti-PD-L1 blocking antibodies are capable of inducing apoptosis of effector PD-L1^+^ CD8^+^ T cells through activating the p38 MAPK pathway, and are thus ineffective as tumor treatments (42). Since this potential agonist effect of anti-mouse PD-L1 antibody was observed in preclinical models, whether therapeutic antibodies to human PD-L1 would have similar agonist effects warrants further investigation.

## The Role of PD-L1 Expressed by Tumor Cells and Tumor-Associated Suppressor Cells

Effector CD8^+^ T cells primed against tumor antigens have the potential to eliminate tumor cells, but have failed to do so in patients with clinically diagnosed cancer. The tumor microenvironment is inhospitable to effector CD8^+^ T cells with numerous overlapping mechanisms in place to inhibit CD8^+^ T cell responses, including the cell-surface expression of PD-L1 by many tumor types (63). Some tumor cells intrinsically express cell-surface PD-L1 while other tumor cells express PD-L1 in response to inflammatory cytokines, a mechanism termed adaptive resistance (64). If a tumor cell that expresses PD-L1 on its surface encounters antitumor CD8^+^ effector T cells that expresses PD-1 or CD80, then the tumor cell will initiate signaling downstream of PD-1 and CD80. Here, we will discuss the outcomes of this encounter.

Effector CD8^+^ T cells isolated from tumor microenvironments exhibit an exhausted phenotype characterized by high surface expression of PD-1 and impaired responses to antigenic stimuli (65–68). It has recently been determined that PD-1 signaling does not initiate differentiation of the exhausted phenotype in CD8^+^ T cells (69), but PD-1 signaling is widely accepted to be the most important receptor involved in maintaining the exhausted phenotype (70). Most of the studies regarding PD-L1/PD-1 signaling in CD8^+^ T cell exhaustion have been done using models of chronic viral infection, but these findings are applicable to tumor settings. Blocking PD-1 signaling has been shown in several models of chronic viral infections to restore antigenic sensitivity of exhausted CD8^+^ T cells. Barber et al. infected mice with lymphocytic choriomeningitis virus clone 13 then treated mice with anti-PD-L1 antibody 23–37 days postinfection. They found significantly decreased viral loads in mice treated with anti-PD-L1 and increased levels of IFN-γ production by CD8^+^ T cells in response to viral antigen stimulation (71). These results were quickly corroborated in HIV, Hepatitis C virus (HCV), and Hepatitis B virus infections (72–78). Blocking PD-L1 signaling in mouse tumor models has also been shown to restore effector antitumor CD8^+^ T cell responses leading to tumor regression and durable antitumor protection (40, 79, 80). Tumor cells also produce a sPD-L1 that can be detected in the plasma of cancer patients and suppresses T cell functions (27). Recently, the increase of sPD-L1 in melanoma patients has been proposed as a potential mechanism of resistance to immune checkpoint blockade therapy (29, 81).

In addition to restraining effector functions in CD8^+^ T cells, PD-L1 signaling also induces apoptosis of effector CD8^+^ T cells (2, 70, 73). Signaling downstream of PD-1 has been shown to inhibit the CD28-mediated upregulation of the pro-survival molecule Bcl-xL (8). HIV-specific CD8^+^ T cells have been shown to express decreased levels of Bcl-xL and exhibit increased susceptibility to apoptosis that is correlated to increased levels of PD-1 surface expression (73, 82). PD-L1 co-stimulation induced increased expression of the pro-apoptotic molecule Bim by effector CD8^+^ T cells in an *in vitro* model (11). Accordingly, PD-L1 co-stimulation failed to induce apoptosis in Bim-deficient effector CD8^+^ T cells. Larrubia et al. also reported increased Bim expression levels in CD8^+^ T cells with high PD-1 expression levels in patients with chronic HCV infection (83). Additionally, PD-1^+^ CD8^+^ T cells isolated from B16 tumors in mice express higher levels of Bim as compared to PD-1^−^ CD8^+^ T cells isolated from the same tumor (84). This same trend was observed in peripheral-blood tumor-reactive CD8^+^ T cells from melanoma patients. Through *in vitro* PD-L1/PD-1 blockade studies, Dronca et al. demonstrated that the anti-PD-1 antibodies currently used clinically (nivolumab and pembrolizumab) decrease Bim expression levels in human PD-1^+^ CD8^+^ T cells in patients who respond to treatment. Importantly, it was demonstrated that PD-L1 induced increased expression of Bim by signaling through both PD-1 and CD80. If the interaction between either PD-L1 and PD-1 or PD-L1 and CD80 was blocked using antibodies, then the PD-L1-induced increase in Bim levels was lost (11). Related to this, it was found that CD80-deficient CD8^+^ exhibited an enhanced memory response to immunization as compared to wild-type CD8^+^ T cells that were co-transferred into a naïve host, again indicating that PD-L1 signaling through CD80 on CD8^+^ T cells restrains the memory response (11). Blockade of PD-L1 may also blunt pro-survival signal of PD-L1 to tumor cells *per se* since it has been reported that reverse signaling of PD-L1 promotes tumor survival and growth (85, 86).

Effector CD8^+^ T cells require a massive amount of energy to maintain rapid proliferation and effector functions. Naïve CD8^+^ T cells utilize oxidative phosphorylation, but upon activation CD8^+^ T cells make a metabolic switch and primarily rely upon the less efficient metabolic program of glycolysis (87, 88). Since glycolysis is less efficient (there are two ATP molecules produced per molecule of glucose used in glycolysis versus 36 ATP molecules produced per molecule of glucose used in oxidative phosphorylation) activated CD8^+^ T cells depend upon very high rates of glycolysis to meet their energy demands and thus require large amounts of glucose to be available in their surrounding environment. Signaling downstream of CD28 co-stimulation activates PI3K/Akt signaling, leading to increased expression of genes necessary for the high glycolytic rate required by activated CD8^+^ T cells (89). The influence of the tumor microenvironment, which is glucose deficient due to the rapid proliferation and energy demands of tumor cells, on metabolism of antitumor effector CD8^+^ T cells has been the focus of numerous recent studies. Here, we will discuss the influence of PD-L1 signaling on the metabolism of antitumor effector CD8^+^ T cells.

It has long been appreciated that signaling downstream of PD-1 ligation by PD-L1 leads to impaired glycolysis due to PD-1-mediated inhibition of CD28 signaling (8). In a graft-versus-host disease model, Saha et al. demonstrated that wild-type donor T cells in syngeneic PD-L1-deficient hosts exhibited increased rates of glycolysis, indicating that PD-L1 signaling serves to inhibit glycolysis downstream of PI3K/Akt signaling (90). Among the necessary genes for glycolysis is the glucose transporter, Glut1, expressed at very high levels on the surface of activated CD8^+^ T cells (91). Syngeneic CD8^+^ T cells in PD-L1 deficient hosts exhibit significantly increased levels of Glut1 expression on the cell surface (90). Tumor microenvironments, in addition to having limited glucose, often have high levels of PD-L1 expression, both of which impair glycolysis in effector CD8^+^ T cells (92). In a mouse model of solid tumors, when PD-L1/PD-1 signaling was blocked, intratumoral CD8^+^ T cells regained their ability to perform glycolysis and effector functions, which led to tumor regression (93). These data indicate that PD-L1/PD-1 checkpoint blockade therapies, in addition to lifting inhibition of effector function of antitumor CD8^+^ T cells, additionally contribute to the regaining of metabolic fitness by antitumor CD8^+^ T cells.

In addition to the priming phase of T cell responses, antigen-presenting cells regulate effector T cell function at peripheral tissues, including inside tumors. Blood monocyte-derived myeloid dendritic cells have cell-surface PD-L1, the expression of which is increased by the hypoxic tumor microenvironment (94). Hypoxia-inducible factor-1α caused a rapid, dramatic, and selective upregulation of PD-L1 on splenic myeloid-derived suppressor cells (MDSCs) along with macrophages, dendritic cells, and tumor cells (95). Among the subsets of MDSC, it seems monocytic-MDSCs (CD14^+^HLA-DR^low^) express higher PD-L1 than other subsets in patients with the diffuse large B-cell lymphoma (96). PD-L1 expressed by tumor-associated MDSCs and macrophages provides an immunosuppressive environment that fosters cancer progression, and blockade of PD-L1 restored the function of tumor-reactive T cells (97). Accordingly, a combination of checkpoint blockade therapy with reagents that inactivate MDSCs is effective in overcoming resistance to checkpoint blockade therapy (98). PD-1 is expressed by tumor-associated macrophages, and signaling downstream of the PD-L1/PD-1 interaction inhibits phagocytosis, a crucial step in innate immunity to cancer (99). PD-1 expressed by monocytes induces IL-10 production and impairs T cell activation (100). It is possible that the co-expression of PD-1 and PD-L1 by macrophages and monocytes may form a regulatory circuit across innate and adaptive immune cells.

## Concluding Remarks

Programmed death-ligand 1/PD-1 checkpoint blockade therapies are becoming an increasingly common treatment option for patients with a variety of tumors. As their use becomes more widespread, it continues to be important that we fully understand the mechanism of action of therapeutic antibodies and their target molecules, as well as develop reliable methods to identify patients most likely to benefit from PD-L1/PD-1 checkpoint blockade therapies. When the interaction between PD-L1^+^ tumor cells and PD-1^+^ CD8^+^ T cells, the intended target for PD-L1/PD-1 checkpoint blockade therapies, is disrupted, the result is reinvigoration of the antitumor CD8^+^ T cell response and enhanced CD8^+^ T cell-mediated killing of tumor cells. As interactions between PD-L1 and PD-1 are not limited to the tumor microenvironment, it is important to consider the influence of the PD-L1/PD-1 interaction that occurs in other contexts. Based on the findings reviewed here, it can be concluded that PD-L1/PD-1 checkpoint blockade therapies likely enhance the priming of antitumor CD8^+^ T cells, but may limit the survival of antitumor CD8^+^ T cells by interfering with a CD8^+^ T cell intrinsic pro-survival/antiapoptotic role for PD-L1 signaling. This context-dependent role for the PD-L1/PD-1 interaction may be responsible in part for the unpredictable patient responses to PD-L1/PD-1 checkpoint blockade therapies.

## Author Contributions

RJ and HD conceived the theme of this review and wrote the manuscript.

## Conflict of Interest Statement

The authors declare that the research was conducted in the absence of any commercial or financial relationships that could be construed as a potential conflict of interest.

## Abbreviations

APC, antigen-presenting cells; B7-H1, B7 homolog 1; Bim, Bcl-2-like protein 11; HCV, hepatitis C virus; HIF-1α, hypoxia-inducible factor-1α; LCMV, lymphocytic choriomeningitis virus; MDC, myeloid dendritic cells; MDSC, myeloid-derived suppressor cell; NSCLC, non-small cell lung cancer; PD-L1, programmed death-ligand 1; sPD-L1, soluble PD-L1.

## References

[1] Dong H Zhu G Tamada K Chen L. B7-H1, a third member of the B7 family, co-stimulates T-cell proliferation and interleukin-10 secretion. Nat Med (1999) 5:1365–9. 10.1038/70932 10581077

[2] Dong H Strome SE Salomao DR Tamura H Hirano F Flies DB Tumor-associated B7-H1 promotes T-cell apoptosis: a potential mechanism of immune evasion. Nat Med (2002) 8:793–800. 10.1038/nm0902-1039c 12091876

[3] Yamazaki T Akiba H Iwai H Matsuda H Aoki M Tanno Y Expression of programmed death 1 ligands by murine T cells and APC. J Immunol (2002) 169:5538–45. 10.4049/jimmunol.169.10.5538 12421930

[4] Keir ME Butte MJ Freeman GJ Sharpe AH. PD-1 and its ligands in tolerance and immunity. Annu Rev Immunol (2008) 26:677–704. 10.1146/annurev.immunol.26.021607.090331 18173375 PMC10637733

[5] Dong H Strome SE Matteson EL Moder KG Flies DB Zhu G Costimulating aberrant T cell responses by B7-H1 autoantibodies in rheumatoid arthritis. J Clin Invest (2003) 111:363–70. 10.1172/JCI16015 12569162 PMC151851

[6] Riley JL. PD-1 signaling in primary T cells. Immunol Rev (2009) 229:114–25. 10.1111/j.1600-065X.2009.00767.x 19426218 PMC3424066

[7] Chemnitz JM Parry RV Nichols KE June CH Riley JL. SHP-1 and SHP-2 associate with immunoreceptor tyrosine-based switch motif of programmed death 1 upon primary human T cell stimulation, but only receptor ligation prevents T cell activation. J Immunol (2004) 173:945–54. 10.4049/jimmunol.173.2.945 15240681

[8] Parry RV Chemnitz JM Frauwirth KA Lanfranco AR Braunstein I Kobayashi SV CTLA-4 and PD-1 receptors inhibit T-cell activation by distinct mechanisms. Mol Cell Biol (2005) 25:9543–53. 10.1128/MCB.25.21.9543-9553.2005 16227604 PMC1265804

[9] Butte MJ Keir ME Phamduy TB Sharpe AH Freeman GJ. Programmed death-1 ligand 1 interacts specifically with the B7-1 costimulatory molecule to inhibit T cell responses. Immunity (2007) 27:111–22. 10.1016/j.immuni.2007.05.016 17629517 PMC2707944

[10] Park JJ Omiya R Matsumura Y Sakoda Y Kuramasu A Augustine MM B7-H1/CD80 interaction is required for the induction and maintenance of peripheral T-cell tolerance. Blood (2010) 116:1291–8. 10.1182/blood-2010-01-265975 20472828 PMC2938239

[11] Gibbons RM Liu X Pulko V Harrington SM Krco CJ Kwon ED B7-H1 limits the entry of effector CD8+ T cells to the memory pool by upregulating Bim. Oncoimmunology (2012) 1:1061–73. 10.4161/onci.20850 23170254 PMC3494620

[12] Ribas A Puzanov I Dummer R Schadendorf D Hamid O Robert C Pembrolizumab versus investigator-choice chemotherapy for ipilimumab-refractory melanoma (KEYNOTE-002):a randomised, controlled, phase 2 trial. Lancet Oncol (2015) 16:908–18. 10.1016/S1470-2045(15)00083-2 26115796 PMC9004487

[13] Robert C Long GV Brady B Dutriaux C Maio M Mortier L Nivolumab in previously untreated melanoma without BRAFMutation. N Engl J Med (2015) 372:320–30. 10.1056/NEJMoa1412082 25399552

[14] Garon EB Rizvi NA Hui R Leighl N Balmanoukian AS Eder JP Pembrolizumab for the treatment of non-small-cell lung cancer. N Engl J Med (2015) 372:2018–28. 10.1056/NEJMoa1501824 25891174

[15] Larkin J Chiarion-Sileni V Gonzalez R Grob JJ Cowey CL Lao CD Combined nivolumab and ipilimumab or monotherapy in untreated melanoma. N Engl J Med (2015) 373:23–34. 10.1056/NEJMoa1504030 26027431 PMC5698905

[16] Robert C Schachter J Long GV Arance A Grob JJ Mortier L Pembrolizumab versus ipilimumab in advanced melanoma. N Engl J Med (2015) 372:2521–32. 10.1056/NEJMoa1503093 25891173

[17] Motzer RJ Escudier B McDermott DF George S Hammers HJ Srinivas S Nivolumab versus everolimus in advanced renal-cell carcinoma. N Engl J Med (2015) 373:1803–13. 10.1056/NEJMoa1510665 26406148 PMC5719487

[18] Borghaei H Paz-Ares L Horn L Spigel DR Steins M Ready NE Nivolumab versus docetaxel in advanced nonsquamous non-small-cell lung cancer. N Engl J Med (2015) 373:1627–39. 10.1056/NEJMoa1507643 26412456 PMC5705936

[19] Brahmer J Reckamp KL Baas P Crinò L Eberhardt WEE Poddubskaya E Nivolumab versus docetaxel in advanced squamous-cell non-small-cell lung cancer. N Engl J Med (2015) 373:123–35. 10.1056/NEJMoa1504627 26028407 PMC4681400

[20] Antonia S Goldberg SB Balmanoukian A Chaft JE Sanborn RE Gupta A Safety and antitumour activity of durvalumab plus tremelimumab in non-small cell lung cancer: a multicentre, phase 1b study. Lancet Oncol (2016) 17:299–308. 10.1016/S1470-2045(15)00544-6 26858122 PMC5500167

[21] Rosenberg JE Hoffman-Censits J Powles T van der Heijden MS Balar AV Necchi A Atezolizumab in patients with locally advanced and metastatic urothelial carcinoma who have progressed following treatment with platinum-based chemotherapy: a single-arm, multicentre, phase 2 trial. Lancet (2016) 387:1909–20. 10.1016/S0140-6736(16)00561-4 26952546 PMC5480242

[22] Rittmeyer A Barlesi F Waterkamp D Park K Ciardiello F von Pawel J Atezolizumab versus docetaxel in patients with previously treated non-small-cell lung cancer (OAK): a phase 3, open-label, multicentre randomised controlled trial. Lancet (2017) 389:255–65. 10.1016/S0140-6736(16)32517-X 27979383 PMC6886121

[23] Kaufman HL Russell J Hamid O Bhatia S Terheyden P D’Angelo SP Avelumab in patients with chemotherapy-refractorymetastatic Merkel cell carcinoma: a multicentre, single-group, open-label, phase 2 trial. Lancet Oncol (2016) 17:1374–85. 10.1016/S1470-2045(16)30364-3 27592805 PMC5587154

[24] Larkin J Minor D D’Angelo S Neyns B Smylie M Miller WH Jr Overall survival in patients with advanced melanoma who received nivolumab versus investigator’s choice chemotherapy in CheckMate 037: a randomized, controlled, open-label phase III trial. J Clin Oncol (2017) 35:1–7. 10.1200/JCO.2016.71.8023 28671856 PMC6804912

[25] Dronca RS Liu X Harrington SM Chen L Cao S Kottschade LA T cell Bim levels reflect responses to anti-PD-1 cancer therapy. JCI Insight (2016) 1:e86014. 10.1172/jci.insight.86014 27182556 PMC4863706

[26] Kamphorst AO Wieland A Nasti T Yang S Zhang R Barber DL Rescue of exhausted CD8 T cells by PD-1-targeted therapies is CD28-dependent. Science (2017) 355:1423–7. 10.1126/science.aaf0683 28280249 PMC5595217

[27] Frigola X Inman BA Lohse CM Krco CJ Cheville JC Thompson RH Identification of a soluble form of B7-H1 that retains immunosuppressive activity and is associated with aggressive renal cell carcinoma. Clin Cancer Res (2011) 17:1915–23. 10.1158/1078-0432.CCR-10-0250 21355078 PMC3241002

[28] Wang L Wang H Chen H Wang W-D Chen X-Q Geng Q-R Serum levels of soluble programmed death ligand 1 predict treatment response and progression free survival in multiple myeloma. Oncotarget (2015) 6:41228–36. 10.18632/oncotarget.5682 26515600 PMC4747402

[29] Zhou J Mahoney KM Giobbie-Hurder A Zhao F Lee S Liao X Soluble PD-L1 as a biomarker in malignant melanoma treated with checkpoint blockade. Cancer Immunol Res (2017) 5:480–92. 10.1158/2326-6066.CIR-16-0329 28522460 PMC5642913

[30] Le DT Uram JN Wang H Bartlett BR Kemberling H Eyring AD PD-1 blockade in tumors with mismatch-repair deficiency. N Engl J Med (2015) 372:2509–20. 10.1056/NEJMoa1500596 26028255 PMC4481136

[31] Herbst RS Soria J-C Kowanetz M Fine GD Hamid O Gordon MS Predictive correlates of response to the anti-PD-L1 antibody MPDL3280A in cancer patients. Nature (2014) 515:563–7. 10.1038/nature14011 25428504 PMC4836193

[32] Tumeh PC Harview CL Yearley JH Shintaku IP Taylor EJM Robert L PD-1 blockade induces responses by inhibiting adaptive immune resistance. Nature (2014) 515:568–71. 10.1038/nature13954 25428505 PMC4246418

[33] Taube JM Klein A Brahmer JR Xu H Pan X Kim JH Association of PD-1, PD-1 ligands, and other features of the tumor immune microenvironment with response to anti-PD-1 therapy. Clin Cancer Res (2014) 20:5064–74. 10.1158/1078-0432.CCR-13-3271 24714771 PMC4185001

[34] Mansfield AS Murphy SJ Peikert T Yi ES Vasmatzis G Wigle DA Heterogeneity of programmed cell death ligand 1 expression in multifocal lung cancer. Clin Cancer Res (2016) 22:2177–82. 10.1158/1078-0432.CCR-15-2246 26667490 PMC4854782

[35] Mansfield AS Aubry MC Moser JC Harrington SM Dronca RS Park SS Temporal and spatial discordance of programmed cell death-ligand 1 expression and lymphocyte tumor infiltration between paired primary lesions and brain metastases in lung cancer. Ann Oncol (2016) 27:1953–8. 10.1093/annonc/mdw289 27502709 PMC5035793

[36] Lau J Cheung J Navarro A Lianoglou S Haley B Totpal K Tumour and host cell PD-L1 is required to mediate suppression of anti-tumour immunity in mice. Nat Commun (2017) 8:1–11. 10.1038/ncomms14572 28220772 PMC5321797

[37] Juneja VR McGuire KA Manguso RT LaFleur MW Collins N Haining WN PD-L1 on tumor cells is sufficient for immune evasion in immunogenic tumors and inhibits CD8 T cell cytotoxicity. J Exp Med (2017) 214:895–904. 10.1084/jem.20160801 28302645 PMC5379970

[38] Masopust D Kaech SM Wherry EJ Ahmed R. The role of programming in memory T-cell development. Curr Opin Immunol (2004) 16:217–25. 10.1016/j.coi.2004.02.005 15023416

[39] Chen L Co-inhibitory molecules of the B7–CD28 family in the control of T-cell immunity. Nat Rev Immunol (2004) 4:336–47. 10.1038/nri1349 15122199

[40] Pulko V Liu X Krco CJ Harris KJ Frigola X Kwon ED TLR3-stimulated dendritic cells up-regulate B7-H1 expression and influence the magnitude of CD8 T cell responses to tumor vaccination. J Immunol (2009) 183:3634–41. 10.4049/jimmunol.0900974 19710456 PMC2789393

[41] Pulko V Harris KJ Liu X Gibbons RM Harrington SM Krco CJ B7-H1 expressed by activated CD8 T cells is essential for their survival. J Immunol (2011) 187(11):5606–14. 10.4049/jimmunol.1003976 22025548 PMC3221917

[42] Liu X Wu X Cao S Harrington SM Yin P Mansfield AS B7-H1 antibodies lose antitumor activity due to activation of p38 MAPK that leads to apoptosis of tumor-reactive CD8. Sci Rep (2016) 6:36722 10.1038/srep36722 27824138 PMC5099859

[43] Freeman GJ Long AJ Iwai Y Bourque K Chernova T Nishimura H Engagement of the PD-1 immunoinhibitory receptor by a novel B7 family member leads to negative regulation of lymphocyte activation. J Exp Med (2000) 192:1027–34. 10.1084/jem.192.7.1027 11015443 PMC2193311

[44] Goldberg MV Maris CH Hipkiss EL Flies AS Zhen L Tuder RM Role of PD-1 and its ligand, B7-H1, in early fate decisions of CD8 T cells. Blood (2007) 110:186–92. 10.1182/blood-2006-12-062422 17392506 PMC1896112

[45] Gibbons RM Liu X Harrington SM Krco CJ Kwon ED Dong H B7-H1 signaling is integrated during CD8+ T cell priming and restrains effector differentiation. Cancer Immunol Immunother (2014) 63:859–67. 10.1007/s00262-014-1563-6 24893858 PMC4833388

[46] Benedict CA Loewendorf A Garcia Z Blazar BR Janssen EM. Dendritic cell programming by cytomegalovirus stunts naive T cell responses via the PD-L1/PD-1 pathway. J Immunol (2008) 180:4836–47. 10.4049/jimmunol.180.7.4836 18354207 PMC2637915

[47] Channappanavar R Twardy BS Suvas S. Blocking of PDL-1 interaction enhances primary and secondary CD8 T cell response to herpes simplex virus-1 infection. PLoS One (2012) 7:e39757. 10.1371/journal.pone.0039757 22808056 PMC3395688

[48] Lanzavecchia A Sallusto F. Progressive differentiation and selection of the fittest in the immune response. Nat Rev Immunol (2002) 2:982–7. 10.1038/nri959 12461571

[49] Mescher MF Curtsinger JM Agarwal P Casey KA Gerner M Hammerbeck CD Signals required for programming effector and memory development by CD8+ T cells. Immunol Rev (2006) 211:81–92. 10.1111/j.0105-2896.2006.00382.x 16824119

[50] Karwacz K Bricogne C MacDonald D Arce F Bennett CL Collins M PD-L1 co-stimulation contributes to ligand-induced T cell receptor down-modulation on CD8+ T cells. EMBO Mol Med (2011) 3:581–92. 10.1002/emmm.201100165 21739608 PMC3191120

[51] Kaech SM Ahmed R Memory CD8+ T cell differentiation: initial antigen encounter triggers a developmental program in naïve cells. Nat Immunol (2001) 2:415–22. 10.1038/87720 11323695 PMC3760150

[52] Schönrich G Kalinke U Momburg F Malissen M Schmitt-Verhulst AM Malissen B Down-regulation of T cell receptors on self-reactive T cells as a novel mechanism for extrathymic tolerance induction. Cell (1991) 65:293–304. 10.1016/0092-8674(91)90163-S 1849799

[53] San José E Borroto A Niedergang F Alcover A Alarcón B. Triggering the TCR complex causes the downregulation of nonengaged receptors by a signal transduction-dependent mechanism. Immunity (2000) 12:161–70. 10.1016/S1074-7613(00)80169-7 10714682

[54] Bachmaier K Krawczyk C Kozieradzki I Kong YY Sasaki T Oliveira-dos-Santos A Negative regulation of lymphocyte activation and autoimmunity by the molecular adaptor Cbl-b. Nature (2000) 403:211–6. 10.1038/35003228 10646608

[55] Chiang YJ Kole HK Brown K Naramura M Fukuhara S Hu RJ Cbl-b regulates the CD28 dependence of T-cell activation. Nature (2000) 403:216–20. 10.1038/35003235 10646609

[56] Naramura M Jang I-K Kole H Huang F Haines D Gu H. c-Cbl and Cbl-b regulate T cell responsiveness by promoting ligand-induced TCR down-modulation. Nat Immunol (2002) 3:1192–9. 10.1038/ni855 12415267

[57] Frigola X Inman BA Krco CJ Liu X Harrington SM Bulur PA Immunology letters. Immunol Lett (2012) 142:78–82. 10.1016/j.imlet.2011.11.001 22138406 PMC3901160

[58] Saha A O’Connor RS Thangavelu G Lovitch SB Dandamudi DB Wilson CB Programmed death ligand-1 expression on donor T cells drives graft-versus-host disease lethality. J Clin Invest (2016) 126:2642–60. 10.1172/JCI85796 27294527 PMC4922691

[59] Ni X Song Q Cassady K Deng R Jin H Zhang M PD-L1 interacts with CD80 to regulate graft-versus-leukemia activity of donor CD8+ T cells. J Clin Invest (2017) 127:1960–77. 10.1172/JCI91138 28414296 PMC5409099

[60] Rowe JH Johanns TM Ertelt JM Way SS. PDL-1 blockade impedes T cell expansion and protective immunity primed by attenuated *Listeria monocytogenes*. J Immunol (2008) 180:7553–7. 10.4049/jimmunol.180.11.7553 18490756 PMC2677094

[61] Seo S-K Jeong H-Y Park S-G Lee S-W Choi I-W Chen L Blockade of endogenous B7-H1 suppresses antibacterial protection after primary *Listeria monocytogenes* infection. Immunology (2008) 123:90–9. 10.1111/j.1365-2567.2007.02708.x 17971153 PMC2433284

[62] Xu D Fu H-H Obar JJ Park J-J Tamada K Yagita H A potential new pathway for PD-L1 costimulation of the CD8-T cell response to *Listeria monocytogenes* infection. PLoS One (2013) 8:e56539. 10.1371/journal.pone.0056539 23409193 PMC3569435

[63] Zou W Chen L. Inhibitory B7-family molecules in the tumour microenvironment. Nat Rev Immunol (2008) 8:467–77. 10.1038/nri2326 18500231

[64] Taube JM Anders RA Young GD Xu H Sharma R McMiller TL Colocalization of inflammatory response with B7-h1 expression in human melanocytic lesions supports an adaptive resistance mechanism of immune escape. Sci Transl Med (2012) 4:127ra37. 10.1126/scitranslmed.3003689 22461641 PMC3568523

[65] Ahmadzadeh M Johnson LA Heemskerk B Wunderlich JR Dudley ME White DE Tumor antigen-specific CD8 T cells infiltrating the tumor express high levels of PD-1 and are functionally impaired. Blood (2009) 114:1537–44. 10.1182/blood-2008-12-195792 19423728 PMC2927090

[66] Sfanos KS Bruno TC Meeker AK De Marzo AM Isaacs WB Drake CG Human prostate-infiltrating CD8 +T lymphocytes are oligoclonal and PD-1. Prostate (2009) 69:1694–703. 10.1002/pros.21020 19670224 PMC2782577

[67] Baitsch L Baumgaertner P Devêvre E Raghav SK Legat A Barba L Exhaustion of tumor-specific CD8+ T cells in metastases from melanoma patients. J Clin Invest (2011) 121:2350–60. 10.1172/JCI46102 21555851 PMC3104769

[68] Fourcade J Sun Z Benallaoua M Guillaume P Luescher IF Sander C Upregulation of Tim-3 and PD-1 expression is associated with tumor antigen-specific CD8+ T cell dysfunction in melanoma patients. J Exp Med (2010) 207:2175–86. 10.1084/jem.20100637 20819923 PMC2947081

[69] Odorizzi PM Pauken KE Paley MA Sharpe A Wherry EJ. Genetic absence of PD-1 promotes accumulation of terminally differentiated exhausted CD8+ T cells. J Exp Med (2015) 212:1125–37. 10.1084/jem.20142237 26034050 PMC4493417

[70] Wherry EJ T cell exhaustion. Nat Immunol (2011) 131:492–9. 10.1038/ni.2035 21739672

[71] Barber DL Wherry EJ Masopust D Zhu B Allison JP Sharpe AH Restoring function in exhausted CD8 T cells during chronic viral infection. Nature (2006) 439:682–7. 10.1038/nature04444 16382236

[72] Day CL Kaufmann DE Kiepiela P Brown JA Moodley ES Reddy S PD-1 expression on HIV-specific T cells is associated with T-cell exhaustion and disease progression. Nature (2006) 443:350–4. 10.1038/nature05115 16921384

[73] Petrovas C Casazza JP Brenchley JM Price DA Gostick E Adams WC PD-1 is a regulator of virus-specific CD8+ T cell survival in HIV infection. J Exp Med (2006) 203:2281–92. 10.1084/jem.20061496 16954372 PMC2118095

[74] Trautmann L Janbazian L Chomont N Said EA Gimmig S Bessette B Upregulation of PD-1 expression on HIV-specific CD8+ T cells leads to reversible immune dysfunction. Nat Med (2006) 12:1198–202. 10.1038/nm1106-1329b 16917489

[75] Zhang J-Y Zhang Z Wang X Fu J-L Yao J Jiao Y PD-1 up-regulation is correlated with HIV-specific memory CD8+ T-cell exhaustion in typical progressors but not in long-term nonprogressors. Blood (2007) 109:4671–8. 10.1182/blood-2006-09-044826 17272504

[76] Urbani S Amadei B Tola D Massari M Schivazappa S Missale G PD-1 expression in acute hepatitis C virus (HCV) infection is associated with HCV-specific CD8 exhaustion. J Virol (2006) 80:11398–403. 10.1128/JVI.01177-06 16956940 PMC1642188

[77] Radziewicz H Ibegbu CC Fernandez ML Workowski KA Obideen K Wehbi M Liver-infiltrating lymphocytes in chronic human hepatitis C virus infection display an exhausted phenotype with high levels of PD-1 and low levels of CD127 expression. J Virol (2007) 81:2545–53. 10.1128/JVI.02021-06 17182670 PMC1865979

[78] Boni C Fisicaro P Valdatta C Amadei B Di Vincenzo P Giuberti T Characterization of hepatitis B virus (HBV)-specific T-cell dysfunction in chronic HBV infection. J Virol (2007) 81:4215–25. 10.1128/JVI.02844-06 17287266 PMC1866111

[79] Blank C Brown I Peterson AC Spiotto M Iwai Y Honjo T PD-L1/B7H-1 inhibits the effector phase of tumor rejection by T cell receptor (TCR) transgenic CD8+ T cells. Cancer Res (2004) 64:1140–5. 10.1158/0008-5472.CAN-03-3259 14871849

[80] Hirano F Kaneko K Tamura H Dong H Wang S Ichikawa M Blockade of B7-H1 and PD-1 by monoclonal antibodies potentiates cancer therapeutic immunity. Cancer Res (2005) 65:1089–96. 15705911

[81] Dronca RS Mansfield AS Liu X Harrington S Enninga EA Kottschade LA Bim and soluble PD-L1 (sPD-L1) as predictive biomarkers of response to anti-PD-1 therapy in patients with melanoma and lung carcinoma. J Clin Oncol (2017) 35(suppl):abstr 11534.

[82] Petrovas C Mueller YM Dimitriou ID Bojczuk PM Mounzer KC Witek J HIV-specific CD8+ T cells exhibit markedly reduced levels of Bcl-2 and Bcl-xL. J Immunol (2004) 172:4444–53. 10.4049/jimmunol.172.7.4444 15034060

[83] Larrubia JR Benito-Martínez S Miquel J Calvino M Sanz-de-Villalobos E González-Praetorius A Bim-mediated apoptosis and PD-1/PD-L1 pathway impair reactivity of PD1+/CD127− HCV-specific CD8+ cells targeting the virus in chronic hepatitis C virus infection. Cell Immunol (2011) 269:104–14. 10.1016/j.cellimm.2011.03.011 21481848

[84] Dronca RS Mansfield AS Park SS Dong H BCL-2-interacting mediator of cell death (Bim) is a novel biomarker for response to anti-PD-1 therapy in patients with advanced melanoma. Immunotherapy (2016) 8:1351–3. 10.2217/imt-2016-0100 27784186

[85] Azuma T Yao S Zhu G Flies AS Flies SJ Chen L. B7-H1 is a ubiquitous antiapoptotic receptor on cancer cells. Blood (2008) 111:3635–43. 10.1182/blood-2007-11-123141 18223165 PMC2275025

[86] Clark CA Gupta HB Curiel TJ. Tumor cell-intrinsic CD274/PD-L1: a novel metabolic balancing act with clinical potential. Autophagy (2017) 13:987–8. 10.1080/15548627.2017.1280223 28368722 PMC5446070

[87] Cham CM Driessens G O’Keefe JP Gajewski TF. Glucose deprivation inhibits multiple key gene expression events and effector functions in CD8+ T cells. Eur J Immunol (2008) 38:2438–50. 10.1002/eji.200838289 18792400 PMC3008428

[88] MacIver NJ Michalek RD Rathmell JC. Metabolic regulation of T lymphocytes. Annu Rev Immunol (2013) 31:259–83. 10.1146/annurev-immunol-032712-095956 23298210 PMC3606674

[89] Frauwirth KA Riley JL Harris MH Parry RV Rathmell JC Plas DR The CD28 signaling pathway regulates glucose metabolism. Immunity (2002) 16:769–77. 10.1016/S1074-7613(02)00323-0 12121659

[90] Saha A Aoyama K Taylor PA Koehn BH Veenstra RG Panoskaltsis-Mortari A Host programmed death ligand 1 is dominant over programmed death ligand 2 expression in regulating graft-versus-host disease lethality. Blood (2013) 122:3062–73. 10.1182/blood-2013-05-500801 24030385 PMC3811178

[91] MacIver NJ Jacobs SR Wieman HL Wofford JA Coloff JL Rathmell JC. Glucose metabolism in lymphocytes is a regulated process with significant effects on immune cell function and survival. J Leukoc Biol (2008) 84:949–57. 10.1189/jlb.0108024 18577716 PMC2638731

[92] Siska PJ Rathmell JC T cell metabolic fitness in antitumorimmunity. Trends Immunol (2015) 36:257–64. 10.1016/j.it.2015.02.007 25773310 PMC4393792

[93] Chang C-H Qiu J O’Sullivan D Buck MD Noguchi T Curtis JD Metabolic competition in the tumor microenvironment is a driver of cancer progression. Cell (2015) 162:1229–41. 10.1016/j.cell.2015.08.016 26321679 PMC4864363

[94] Curiel TJ Wei S Dong H Alvarez X Cheng P Mottram P Blockade of B7-H1 improves myeloid dendritic cell–mediated antitumor immunity. Nat Med (2003) 9:562–7. 10.1038/nm863 12704383

[95] Noman MZ Desantis G Janji B Hasmim M Karray S Dessen P PD-L1 is a novel direct target of HIF-1α, and its blockade under hypoxia enhanced MDSC-mediated T cell activation. J Exp Med (2014) 211:781–90. 10.1084/jem.20131916 24778419 PMC4010891

[96] Azzaoui I Uhel F Rossille D Pangault C Dulong J Le Priol J T-cell defect in diffuse large B-cell lymphomas involves expansion of myeloid-derived suppressor cells. Blood (2016) 128:1081–92. 10.1182/blood-2015-08-662783 27338100

[97] Kuang D-M Zhao Q Peng C Xu J Zhang J-P Wu C Activated monocytes in peritumoral stroma of hepatocellular carcinoma foster immune privilege and disease progression through PD-L1. J Exp Med (2009) 206:1327–37. 10.1084/jem.20082173 19451266 PMC2715058

[98] Lu X Horner JW Paul E Shang X Troncoso P Deng P Effective combinatorial immunotherapy for castration-resistant prostate cancer. Nature (2017) 543:728–32. 10.1038/nature21676 28321130 PMC5374023

[99] Gordon SR Maute RL Dulken BW Hutter G George BM McCracken MN PD-1 expression by tumour-associated macrophages inhibits phagocytosis and tumour immunity. Nature (2017) 545:495–9. 10.1038/nature22396 28514441 PMC5931375

[100] Said EA Dupuy FP Trautmann L Zhang Y Shi Y El-Far M Programmed death-1–induced interleukin-10 production by monocytes impairs CD4+ T cell activation during HIV infection. Nat Med (2010) 16:452–9. 10.1038/nm.2106 20208540 PMC4229134

